# Body adiposity indices are associated with hypertension in a black, urban Free State community

**DOI:** 10.4102/phcfm.v6i1.581

**Published:** 2014-05-19

**Authors:** Ronette Lategan, Violet L. van den Berg, Corinna M. Walsh

**Affiliations:** 1 Faculty of Health Sciences, University of the Free State, South Africa

## Abstract

**Background:**

Non-communicable diseases, including hypertension, are increasing rapidly in resource-poor, developing countries amongst populations transitioning from traditional to westernised lifestyles; and are associated with excess weight.

**Aim:**

To investigate the relationship between hypertension and various indices of body adiposity in a transitioning, urban, black population.

**Setting:**

Three hundred and thirty-nine adults (25–64 years) from a larger cross-sectional study (Assuring Health for All in the Free State) conducted in Mangaung, South Africa, were included.

**Methods:**

Standard techniques were used to determine blood pressure, HIV status, body mass index (BMI), waist-to-height ratio (WHtR) and body adiposity index (BAI).

**Results:**

Approximately 40% of the sample was HIV-positive and 63.4% hypertensive, with the greatest risk of hypertension being amongst older men. Based on BMI, 23.0% were overweight and 32.1% obese. Waist-to-height ratio showed that 58.6% had increased cardiovascular risk. Mean BAI was 34.1%, whilst 76.3% had a body fat percentage in the overweight/obese category. Waist circumference representing increased cardiovascular risk was found in 44.3% of women and 3.9% of men. Significant positive correlations between mean arterial blood pressure and BMI (*r* = 0.261; *p* < 0.001), WHtR (*r* = 0.357; *p* < 0.001) and BAI (*r* = 0.245; *p* < 0.001) were found. WHtR was a stronger predictor of mean arterial pressure than BMI or BAI. HIV status showed an inverse correlation with all adiposity indices (*p* < 0.001).

**Conclusion:**

Our findings promote WHtR as a practical screening tool for increased hypertension risk in populations undergoing westernisation, and support weight loss as a first-line intervention for the prevention and management of hypertension.

## Abstrait

**Les indices d'adiposité corporelle sont associés à l'hypertension dans une communauté africaine et urbaine de la province de l'Etat libre**.

**Présentation:**

Les maladies non transmissibles, notamment l'hypertension, connaissent une augmentation rapide dans les pays aux ressources limitées et en voie de développement parmi les populations en transition entre un style de vie traditionnel et un style de vie occidentalisé; elles sont également associées à un excédent de poids.

**Objectif:**

Etudier la relation entre l'hypertension et différents indices d'adiposité corporelle dans une population africaine, en transition et urbaine.

**Cadre:**

Trois cent trente-neuf adultes (âgés de 25 à 63 ans) sélectionnés à partir d'une étude transversale de plus grande envergure (Assurer la Santé pour Tous dans l'Etat Libre) réalisée à Mangaung, en Afrique du Sud, ont été inclus.

**Méthodes:**

Des techniques standards ont été utilisées afin de déterminer la tension artérielle, la séropositivité, l'indice de masse corporelle (IMC), le rapport taille-hanche (RTH) et l'indice d'adiposité corporelle (IAC).

**Résultats:**

Environ 40% de l’échantillon étaient séropositifs et 63.4% étaient hypertensifs, le risque d'hypertension le plus élevé étant observé chez les hommes âgés. Sur la base de l'IMC, 23% étaient en surpoids, et 32.1 étaient obèses. Le RTH indiquait que 58.6% présentaient un risque cardiovasculaire accru. L'IAC moyen était de 34.1%, et 76.3% des participants à l’étude avaient un pourcentage de graisse corporelle entrant dans la catégorie surpoids/obèse. La circonférence de la taille représentant un risque cardiovasculaire accru a été mesurée à 44.3% chez les femmes et à 3.9% chez les hommes. Des corrélations significatives entre la tension artérielle moyenne et l'IMC (*r* = 0.261; *p* < 0.001), le RTH (*r* = 0.357; *p* < 0.001) et l'IAC (*r* = 0.245; *p* < 0.001) ont été observées. Le RTH était une variable causale plus importante de la tension artérielle moyenne que l'IMC ou l'IAC. La séropositivité présentait une corrélation inverse avec tous les indices d'adiposité (*p* < 0.001).

**Conclusion:**

Nos conclusions indiquent que le RTH devrait être utilisé comme outil de dépistage pratique du risque d'hypertension accru chez les populations en transition, et que la perte de poids devrait être recommandée comme intervention de première ligne pour la prévention et la gestion de l'hypertension.

## Introduction

Hypertension is a leading cause of morbidity in middle-income countries. It is gaining significance in low-income countries, contributing to an increasing global disease burden.^1, 2^ Underdiagnosis and/or inadequate treatment of hypertension may cause organ damage and premature death,^1, 3^ affecting the economy of a country and making prevention and early intervention important objectives for health authorities worldwide.

It has been estimated that hypertension accounted for 9% of deaths in South Africa in 2000, making it the second-highest cause of mortality after sexually-transmitted diseases.^2^ South Africa has been undergoing a transition from traditional diets and more active lifestyles to typically Western diets and more sedentary practices. These trends are associated with the high prevalence of obesity amongst black South Africans, particularly women in urban areas.^3^

A strong relationship exists between body weight and blood pressure, with the risk of developing hypertension being two to six times higher in overweight than in normal weight individuals.^1^ Overweight and general adiposity are associated independently with an increased risk for heart failure.^4^ A strong relationship also exists between the degree of obesity as measured by body mass index (BMI) and waist circumference (WC), and the prevalence of ischaemic stroke, regardless of gender or race.^5^ Weight loss as a first-line treatment for lowering blood pressure is therefore generally recommended.^1, 3, 6, 7^ It has been found that 5.1 kg of weight loss caused a reduction of 4.44 mmHg and 3.57 mmHg in systolic and diastolic pressure, respectively, translating to a reduction of 1.05 mmHg in systolic and 0.92 mmHg in diastolic pressure per kilogram of weight lost.^7^ Markers of general obesity and indicators of abdominal obesity are good predictors of cardiovascular risk. Waist circumference (WC), waist-to-height ratio (WHtR), BMI and visceral fat percentage are strong predictors of arterial stiffness in morbidly obese female patients.^8^

The World Health Organization (WHO) recognises two thresholds for abdominal obesity as measured by WC, depicting different levels of risk for metabolic (including cardiovascular) complications. Increased risk is at a WC of > 80 cm in women and > 94 cm in men, with substantially increased risk at a WC of > 88 cm for women and > 102 cm for men.^9^ Even a WC within the higher parameters of normal cut-off points is associated with a high prevalence of hypertension in older adults, independent of BMI and other hypertension risk factors.^10^

The risk for metabolic complications occurs at different levels of adiposity (indicated by BMI) and central obesity (indicated by WC) in different populations. It has been recommended that the current BMI cut-off points should be retained as international classification, but that more detail be added for reporting purposes (18.5 kg/m^2^ – 23 kg/m^2^ increasing but acceptable risk; 23 kg/m^2^ – 27.5 kg/m^2^ increased risk; and ≥ 27.5 kg/m^2^ high risk) to facilitate international comparison.^11^ Different ethnicity-specific cut-off points have also been suggested for WC.^12^

As no ethnicity-specific WC recommendations exist for South Africans, the recommendations for sub-Saharan Africa^13, 14^ (80 cm for women; 94 cm for men) can be considered, which are approximately 10% lower than the recommendation for North Americans.^12^

WHtR is regarded as a better and more sensitive obesity marker than BMI for predicting hypertension and as an early marker of metabolic health risks.^15, 16^ An advantage of WHtR is that the same cut-off point (0.5) can be used for both men and women of different ethnic groups.^15^

Body adiposity index (BAI), a direct estimate of percentage body fat calculated from hip circumference and height, is an easily-obtained measure to predict the risk for obesity-related diseases.^17^

Despite strong evidence that supports the association between body weight and hypertension, Schutte et al.^18^ failed to show a strong relationship between obesity markers (BMI and fat percentage) and blood pressure amongst black South African women.

The high prevalence of HIV infection in South Africa should be considered. Hypertension is equally common amongst both HIV-infected and -uninfected individuals. Although multivariate models link increasing age, higher BMI, longer duration of infection and diabetes to hypertension in HIV-infected populations, the prevalence of hypertension is apparently not influenced by HIV status or the use of highly active antiretroviral therapy (HAART).^19, 20, 21, 22, 23^

The Assuring Health for All in the Free State (AHA-FS) study investigated the double burden of infectious diseases and undernutrition, on the one hand, and obesity and its comorbidities on the other, in a low-income, black, urban community in central South Africa. Hypertension occurred in 56.9% (*n* = 415) of the study population and mean systolic and diastolic blood pressure measurements were 135.5 ± 23.9 mmHg and 89.8 ± 17.4 mmHg, respectively.^24^ The high prevalence of hypertension in this population was the motivation for investigating its primary causes. The objective of this substudy, therefore, was to invesitgate to investigate the relationship between blood pressure and various indices of body adiposity (BMI, WC, WHtR and BAI) in a transitioning urban black population, whilst taking into account the influence of HIV.

## Research methods and design

Approval to conduct the study was obtained from the Ethics Committee of the Faculty of Health Sciences, University of the Free State in Bloemfontein, South Africa.

For this cross-sectional study, data from the urban AHA-FS study^24^ were used. Data were collected over a period of two weeks in March 2009 from six areas in Mangaung, South Africa, namely Freedom Square, Turflaagte, Namibia, Kagisanong, Chris Hani and Rocklands. Households were selected by proportional cluster sampling, stratified by area and formal plot/squatter households in open areas. Using randomly selected X- and Y-coordinates, 100 starting points were selected. Trained field workers approached five adjacent households from each starting point in order to obtain written informed consent from eligible adults (25–64 years). In the AHA-FS study, a variety of measurements, including body weight, height, waist and hip circumferences were taken, blood pressure was measured and blood samples were drawn for various biochemical tests. A subsample, with complete data sets for age, gender, blood pressure, body weight, height and HIV status, was included in this study.

Blood pressure was measured by a registered medical practitioner according to recognised guidelines.^25, 26^ Hypertension was defined as systolic blood pressure of ≥ 140 mmHg and/or diastolic blood pressure ≥ 90 mmHg.^1^ Participants using prescription medication for the management of hypertension at the time of the interview were also classified as hypertensive.^25, 26^

HIV status was determined from fasting venous blood samples. Primary screening for HIV was performed using the Enzygnost HIV Integral II Ag/Ab test (Dade Behring, Marburg, Germany) and results were confirmed by the Vironostika HIV Uni-Form II Ag/Ab test (bioMérieux, Marcy l'Etoile, France).

Anthropometric measurements were taken by trained final-year Dietetics students, under supervision of the researchers. Body weight was determined using WHO guidelines^27^ on a floor-type Seca 770 digital scale (Medical Scales and Measuring Systems Seca kk., Japan) with accuracy to 100 g and a maximum capacity of 200 kg. Height was measured^27^ with a Seca stadiometer (Medical Scales and Measuring Systems Seca kk., Japan) accurate to the nearest 5 mm. BMI (kg/m^2^) was calculated and interpreted according to WHO guidelines.^27, 28^ Waist circumference was measured with a non-stretch measuring tape, midway between the top of the superior iliac crest and the lowest rib in the mid-axillary line.^9^

Waist circumference was interpreted according to WHO guidelines^9^ as increased risk of metabolic complications at > 80 cm in women and > 94 cm in men; and as a substantially-increased risk of metabolic complications at a WC > 88 cm in women and > 102 cm in men. WHtR was determined by dividing WC (cm) by height (cm). A WHtR of ≥ 0.5 indicates an increased risk for non-communicable diseases (NCDs) in both genders.^15^

Hip circumference was measured at the widest portion of the buttocks parallel to the floor, accurate to the nearest 5 mm.^27^

Hip circumference and height were used to calculate BAI (see formula below),^17^ which provides an accurate estimate of body fat percentage when compared to dual-energy X-ray absorptiometry (DXA).^17^

BAI (% body fat) = hip circumference (cm) ÷ height (m)^1.5^ – 18. BAI results were interpreted according to the classification proposed by Ricciardi et al.^29^ namely:

- low body fat: 10% – 15% (men); 14% – 20% (women)
- average body fat: 16% – 18% (men); 21% – 25% (women)
- above average body fat: 19% – 20% (men); 26% – 29% (women)
- overweight body fat: 21% – 25% (men); 30% – 35% (women)
- obese body fat: ≥ 26% (men); ≥ 36% (women).

### Statistical analysis

Analysis of data was performed with PASW (Predictive Analytics SoftWare) statistics software by SPSS version 18 (Chicago, SPSS Inc.). Frequencies and percentages were used to express categorical data. Means and standard deviations, or percentiles, as appropriate, were used to express quantitative variables. Comparisons of means were performed using *t*-tests. Chi-square tests, two-tailed Pearson correlations, point bi-serial correlations and multivariate logistic regression models were used to describe and test associations between variables; and relative risk of incidence was calculated.

### Ethical considerations

Ethical clearance was granted by the Ethics Committee, Faculty of Health Sciences, University of the Free State, Bloemfontein, South Africa (ETOVS number 21/07).

## Results

### Age, blood pressure, anthropometry and HIV status

A total of 339 adults (76 men [22.4%]; 263 women [77.6%]) with a mean age of 44.3 years (range 25–64 years) were included in the study. Table 1 summarises the participants’ information in terms of age, blood pressure (systolic, diastolic and mean arterial pressure) and anthropometry.

**TABLE 1:** Age, blood pressure and anthropometric measurements of participants (*N* = 339, unless indicated otherwise).

Variable	Mean	Standard deviation (SD)	Range
**Age**			
Years	44.3	± 10.6	25–64
**Blood pressure (mmHg)**			
*Systolic pressure*			
Total group	135.5	± 23.7	72–203
Men (*n* = 76)	134.8	± 22.8	87–188
Women (*n* = 263)	135.7	± 24.0	72–203
*Diastolic pressure*			
Total group	89.8	± 17.6	46–188
Men (*n* = 76)	86.6	± 15.2	54–119
Women (*n* = 263)	90.7	± 18.1	46–188
*Mean arterial pressure*			
Total group	105.0	± 18.0	59–191
Men ( *n*= 76)	102.7	± 15.6	68–133
Women (*n* = 263)	105.7	± 18.5	59–191
Height (cm)	159.4	± 7.9	139.8–180
Weight (kg)	70.1	± 21.4	31.9–140
**Body mass index (BMI; kg/m^2^)**			
Total group	27.8	± 8.8	13.3–55.7
Men (*n* = 76)	21.4	± 5.6	14.5–49.9
Women (*n* = 263)	29.6	± 8.7	13.3–55.7
**Waist circumference (WC; cm)**			
Men (*n* = 76)	78.1	± 13.9	47–134
Women (*n* = 262)	88.9	± 17.1	59–143
Waist-height ratio (WHtR) (*N* = 338)	0.55	± 0.11	0.27–0.91
Hip circumference (cm) (*N* = 338)	104.5	± 18.2	73.5–165
**Body adiposity index (BAI;%)**			
Men (*n* = 76)	24.1	± 5.2	16.8–50.7
Women (*n* = 262)	37.1	± 9.5	19.2–66.1

More than a third (39.8%; *n* = 135) of the sample was HIV-infected and the mean BMI was significantly lower (*p* < 0.001) in HIV-infected participants (24.9 ± 7.4 kg/m^2^), compared with HIV-uninfected participants (29.6 ± 9.2 kg/m^2^). In 41.6% (*n* = 141) of individuals, systolic blood pressure was ≥ 140 mmHg, whilst 46.6% (*n* = 158) had a diastolic blood pressure of ≥ 90 mmHg, indicating hypertension. Approximately one quarter (25.4%; *n* = 86) of participants used antihypertensive medication at the time of the study. Consequently, 63.4% (57.9% [*n* = 44] men and 65.0% [*n* = 171] women) of the participants were classified as hypertensive.

### Distribution of body fat indices

The distribution of body fat indices of the participants is summarised in Table 2A, B, C and D.

**TABLE 2A:** Body mass index (BMI) according to the categories recommended by the WHO for international classification.^11^

BMI kg/m^2^	Classification	Men (*n* = 76)		Women (*n* = 263)	
	
		*n*	%	*n*	%
< 18.5	Underweight	19	25.0	21	8.0
18.5–24.9	Normal	43	56.6	69	26.2
25.0–29.9	Overweight	11	14.5	67	25.5
30.0–34.9	Obese Class 1	0	0	39	14.8
35.0–39.9	Obese Class 2	2	2.6	32	12.2
≥ 40.0	Obese Class 3	1	1.3	35	13.3

**TABLE 2B:** Waist circumference (WC) according to the categories recommended by the WHO.^9^

Level of risk	Gender-specific cut-offs		Men (*n* = 76)		Women (*n* = 262)	
	
			*n*	%	*n*	%
Low risk	Men:	< 94 cm	71	93.4	87	33.2
	Women	< 80 cm				
Increased risk	Men:	94–102 cm	2	2.6	59	22.5
	Women	80–88 cm				
High risk (central obesity)	Men:	> 102 cm	3	3.9	116	44.3
	Women	> 88 cm				

**TABLE 2C:** Waist-to-height ratio (WHtR).^16^

Level of risk	Universal cut-offs	Men (*n* = 76)		Women (*n* = 262)	
	
		*n*	%	*n*	%
Low risk	< 0.5	61	80.3	79	30.2
At risk	≥ 0.5	15	19.7	183	69.8

**TABLE 2D:** Body adiposity index (BAI) according to body fat% categories.^29^

Body fat (%)	Gender-specific cut-offs		Men (*n*=76)		Women (*n*=263)	
	
			*n*	%	*n*	%
Low	Men:	10–15%	0	0	2	0.8
	Women:	14–20%				
Average	Men:	16–18%	4	5.3	30	11.5
	Women:	21–25%				
Above average	Men:	19–20%	16	21.1	28	10.7
	Women:	26–29%				
Overweight	Men:	21–25%	39	51.3	72	27.4
	Women:	30–35%				
Obese	Men: ≥ 26%		17	22.4	130	49.6
	Women: ≥ 36%					

Based on BMI, 18.4% (*n* = 14) of the men and 65.8% (*n* = 173) of the women were overweight/obese. With regard to WC, using cut-off points for sub-Saharan populations, 6.5% (*n* = 5) of the men (WC > 94 cm) and 66.8% (*n* = 175) of the women (WC > 80 cm) showed increased health risk.

An overweight/obese BMI was associated significantly (χ^2^ = 144.533; *p* < 0.001) with high-risk WC (> 88 cm in women; > 102 cm in men). Based on a WHtR of ≥ 0.5, 19.7% (*n* = 15) of the men and 69.8% (*n* = 183) of the women were at risk. When taking BAI into consideration, 73.7% (*n* = 56) of the men and 77.1% (*n* = 202) of the women had body fat percentages in the overweight/obese category.

### Occurrence of hypertension in relation to age, gender and body fat indices

A major risk factor for increased arterial blood pressure is age. To assess the effect of age, participants were stratified according to mean age into two age groups, namely > 44 years (*n* = 172) and ≤44 years (*n* = 167). Hypertension was significantly more prevalent in the older group (χ^2^ = 45.526; *p* < 0.0001). A relative risk for hypertension of 1.78 (95% confidence interval [CI] 1.48; 2.13) was calculated, indicating that the older group had a 78% higher risk of being hypertensive than the younger group. When genders were compared, controlling for age, the relative risk for hypertension was 2.4 (95% CI 1.47; 3.91) for men and 1.66 (95% CI 1.37; 2.01) for women. The occurrence of hypertension in relation to BMI categories is shown in Table 3.

**TABLE 3:** Distribution of normotensive and hypertensive participants across BMI categories.

Body mass index (BMI)	Normotensive (*n* = 124)		Hypertensive (*n* = 215)	
	
	*n*	%	*n*	%
**Weight**				
Underweight (< 18.5 kg/m^2^)	23	18.5	17	7.9
Normal weight (18.5–24.9 kg/m^2^)	57	46.0	55	25.6
Overweight (25.0–29.9 kg/m^2^)	23	18.5	55	25.6
**Obese**				
Class I (30.0–34.9 kg/m^2^)	5	4.0	34	15.8
Class II (35.0–39.9 kg/m^2^)	6	4.8	28	13.0
Class III (≥ 40 kg/m^2^)	10	8.1	26	12.1

When stratified into low to normal BMI (< 25 kg/m^2^; low risk) and overweight/obese BMI (≥ 25 kg/m^2^; high risk), 76.5% (*n* = 143) of the overweight/obese group had hypertension, which was a significant finding (χ^2^ = 30.611; *p* < 0.0001) in this group. After controlling for age, participants > 44 years with an overweight/obese BMI had a relative risk of 1.25 (95% CI 1.06; 1.46) of being hypertensive, compared with a normal/underweight BMI. In the ≤44 years group, the relative risk of being hypertensive was 2.69 (95% CI 1.75; 4.15) when being overweight/obese versus being normal/underweight.

When applying a multivariate logistic regression model (mean arterial pressure as independent variable and age and BMI as dependent variables), BMI and age accounted for 14.8% of the change in arterial pressure. For every one year increase in age, or every 1 kg/m^2^ increase in BMI, a 0.48 mmHg increase in mean arterial pressure could be expected (*p* < 0.001).

In Table 4, the prevalence of hypertension according to WC categories is summarised, indicating an increased prevalence of hypertension with increasing WC for women (χ^2^ = 32.062; *p* < 0.0001). An independent samples *t*-test confirmed that WC in the hypertensive group was significantly higher than the normotensive group and correlations showed that mean arterial blood pressure correlated significantly with WC in both men (*r* = 0.262; *p* < 0.05) and women (*r* = 0.382; *p* < 0.001).

**TABLE 4:** Waist circumference in relation to the prevalence of hypertension.

Waist circumference category	Men (*n* = 76)				Women (*n* = 262)			
	
	Normotensive (*n* =32)		Hypertensive (*n* = 44)		Normotensive (*n* = 92)		Hypertensive (*n* = 169)	
				
	*n*	%	*n*	%	*n*	%	*n*	%
Lower risk (*n* = 159) (Women: ≤ 88 cm; Men: ≤ 102 cm)	32	42.1	39	51.3	52	19.8	35	13.4
Increased risk (*n* = 61) (Women: 80–88 cm; Men: 94–102 cm)	0	0	2	2.6	21	8.0	38	14.5
Central obesity (*n* = 118) (Women: > 88 cm; Men: > 102 cm)	0	0	3	3.9	19	7.3	97	37.0

When female participants were stratified according to WC as > 88 cm and ≤ 88 cm and controlled for age (< 44 years and ≥ 44 years), older women with high-risk WC had a relative risk of 1.29 (95% CI 1.08; 1.53) of being hypertensive; whilst younger women with high-risk WC had a relative risk of 2.22 (95% CI 1.56; 3.14) of being hypertensive.

Both WHtR *(r* = 0.357; *p* < 0.001) and BAI (*r* = 0.245; *p* < 0.001) correlated significantly with mean arterial blood pressure. A significant relation was found between a WHtR of ≥ 0.5 (increased risk) and the prevalence of hypertension (χ^2^ = 43.057; *p* < 0.001). WHtR (*r* = 0.357) showed a stronger correlation with mean arterial pressure than BMI (*r* = 0.261) or BAI (*r* = 0.245).

### HIV status and blood pressure

Mean arterial pressure was correlated inversely to HIV status (*r* = -0.217; *p* < 0.001). However, partial correlation indicated that this inverse association resulted from the effect of age and adiposity levels on blood pressure. When using a point bi-serial correlation, HIV status showed an inverse correlation with BMI (*r* = -0.262; *p* < 0.001), WC (*r* = -0.364; *p* < 0.001), WHtR (*r* = -0.299; *p* < 0.001), and BAI (*r* = -0.226; *p* < 0.001) in women.

## Discussion

The prevalence of hypertension, which affected 63.4% of the total group in this low-income, urban, black population, compares with 61.8% reported for an African-American population older than 40 years.^30^ The high prevalence of hypertension, especially amongst a population that included younger participants, raises concern.

More than half (55.1%; *n* = 187) of the participants had BMIs above normal (23.0% overweight; 32.1% obese). This is higher than the WHO estimate of 45.1% for the prevalence of overweight/obesity in the general South African population^27^ and the 48% reported for South Africans from 18 companies participating in a health-risk screening programme.^31^ For men, the prevalence of overweight/obesity was lower (18.4%) than the prevalence described for Free State men in the South African National Health and Nutrition Examination Survey study (25.3%), but for women, the prevalence was higher (65.8% vs 63.7%).^32^ The mean BMI for this study (27.8 kg/m^2^) was lower than the mean of 29.8 ± 0.2 kg/m^2^ for Americans of combined races,^30^ as well as the mean of 30.1 kg/m^2^ in African-Americans as reported previously.^33^

With regard to above-normal BMIs, 14.5% (*n* = 11) and 3.9% (*n* = 3) of men (mean BMI 21.4 kg/m^2^) and 25.5% (*n* = 67) and 40.3% (*n* = 106) of women (mean BMI 29.6 kg/m^2^) were overweight and obese, respectively. This finding corresponds with data from the South African Demographic and Health Survey,^34^ reporting 18.7% of urban black men as overweight and 8.1% as obese (mean BMI 23.1 kg/m^2^) and 27.1% of urban black women as overweight and 33.8% as obese (mean BMI 28.1 kg/m^2^). Our findings confirmed the trend that black South African women have substantially higher BMIs than their male counterparts, as reported previously. Charlton et al.^35^ reported a mean BMI of 28 kg/m^2^ in black South African men (37.7% obese) and 33.5 kg/m^2^ in black South African women (67.9% obese) in Cape Town. The Transition and Health During Urbanisation of South Africans (THUSA) study^36^ reported that 28.6% of black South African female participants were obese and 53.8% had a BMI > 25 kg/m^2^. Contrary to the higher BMIs found amongst women in South Africa, a mean BMI of 26.4 kg/m^2^ for American men and 26.2 kg/m^2^ for women have been reported.^37^

The high prevalence of overweight/obesity in this population, linked to the prevalence of hypertension, agrees with the International Study of Salt and Blood Pressure (INTERSALT),^38^ reporting a strong, significant, independent association of BMI with blood pressure.

BMI in this study was related significantly (*p* < 0.001) to to WC. Black South African women had a similar mean WC (88.9 cm) than American women (88.5 cm).^37^ In contrast, black South African men in our study had a lower mean WC (78.1 cm) than American men (95.6 cm). The incidence of high-risk WC was higher than the national data for urban black South Africans^34^ (3.9% versus 3.1% for men; and 44.3% versus 39.1% for women). Charlton et al.^35^ also described a higher mean WC in a black population, namely 92.6 cm in men and 92.4 cm in women.

Mashele et al.^39^ found that, similar to our findings, urbanised hypertensive black South African men and women were typically older, more obese and had a larger WC compared with their normotensive counterparts.

Based on WHtR and BAI as obesity indices, a high percentage of participants in the current study were at increased risk for NCD. WHtR was increased in 58.6% (*n* = 198) of participants, whilst 76.3% (*n* = 258) of all participants had a BAI in the overweight/obese category. We found a slightly stronger association of hypertension with WHtR than with BMI and BAI, although all three indices were associated significantly with mean arterial blood pressure. These findings concur with a meta-analysis^40^ reporting that measures of abdominal obesity were better predictors of cardiovascular disease risk than BMI. More importantly, however, WHtR could be seen as a simple tool to screen for hypertension in this particular community. At a primary healthcare level, the community could be taught to use a piece of string equal to their height; if the string cannot go around the waist at least twice, the risk for hypertension and other non-communicable diseases is reflected.

Almost 40% (*n* = 135) of participants in our study were infected with HIV. Although the prevalence of hypertension showed an inverse association with HIV status, this association was found to result from the lower body adiposity as represented by lower BMI, WHtR, BAI and WC, which are associated with HIV infection. A similar lack of significant associations between hypertension and HIV status has been reported previously.^19, 20, 21, 22, 23^

A degree of bias is acknowledged. More women may have participated because the research was conducted during a week day, resulting in less men attending because of being formally employed. More ill people may have participated due to free medical examinations being conducted during the study.

## Conclusion

Obesity, hypertension and HIV occurred commonly amongst participants in this study. The prevalence of hypertension was not influenced significantly by HIV status when adiposity was taken into account. WHtR was related significantly to mean arterial pressure and seemed to be a stronger predictor of mean arterial pressure than BMI or BAI. Obesity was more prevalent in women than men; and, in women, hypertension was related significantly to WC and BMI. These results support weight loss as the first line of intervention for the treatment and prevention of hypertension with its accompanying disease burden. We propose that WHtR may be an easy and practical way of screening for hypertension and motivating communities to decrease their waist circumference. Future research to assess the impact of a simple nutrition message, ‘Stay healthy by keeping your waist circumference less than half of your height’, is recommended.
